# Investigating the Role of Wearable Devices in Facilitating Telehealth Adoption Among the Aging Population: Mediation Analysis of US National Data

**DOI:** 10.2196/68559

**Published:** 2025-08-07

**Authors:** Ruijing Wang, Onur Asan, Ting Liao

**Affiliations:** 1 Department of Systems Engineering, Stevens Institute of Technology, 1 Castle Point Terrace, Hoboken, NJ, 07030, United States, 1 (201) 2168643

**Keywords:** telehealth, health outcomes, wearable devices, aging population, mediation analysis

## Abstract

**Background:**

Telehealth adoption has grown significantly, presenting valuable opportunities for the aging population to access health care remotely. Despite evidence of its benefits in managing chronic conditions and promoting independence, many older adults remain hesitant to adopt telehealth, preferring traditional in-person visits even post pandemic. Current literature largely focuses on younger or general populations, overlooking the unique barriers faced by older adults, such as technology literacy and access disparities.

**Objective:**

This study investigates how telehealth adoption among the aging population is influenced and mediated by relevant factors, including the use of wearable devices, demographic factors, health conditions, and physical activity levels.

**Methods:**

A secondary analysis was conducted on the Health Information National Trends Survey (HINTS 6) data collected from March to November 2022. Of the 6252 respondents, 1596 older adults (≥65 years) were included. Telehealth adoption was defined using 2 survey items on receiving or being offered telehealth services. We construct regression and mediation analyses to understand the relationships between telehealth adoption and influential factors, including demographics, physical activity levels, health conditions, and the use of wearable devices.

**Results:**

We found that wearable device use, while not directly significant, plays a critical role in adoption when mediated by factors such as education, income, and general health. Specifically, higher levels of education and income increased the likelihood of telehealth adoption (*P*<.001), underscoring the importance of socioeconomic status. Additionally, rural versus urban residency emerged as a critical factor (*P*=.003), with rural residents demonstrating lower adoption rates, highlighting the accessibility and technology literacy barriers in these areas. Health conditions were inversely associated with telehealth adoption, suggesting that healthier individuals may perceive less need for telehealth services. The total effect of wearable use on telehealth adoption was significant (*P*=.007), with indirect effects via education (*P*<.001), income (*P*=.007), and health conditions (*P*=.004). The findings underscore the role of socioeconomic factors in influencing the adoption of health technologies.

**Conclusions:**

While wearable device use is associated with increased telehealth adoption among older adults, its effect operates primarily through mediating factors such as education, income, and health status. These findings suggest that addressing disparities in socioeconomic status and health literacy is critical to increasing telehealth engagement in aging populations.

## Introduction

### Background

The health care system is under growing pressure, including an aging population, long-term health conditions, and increasingly higher standards of care expected by society, government, patients, and professionals [1]. The role of technology in health care has become increasingly important, giving rise to the concepts of telehealth and telemedicine as key components of modern health care systems [2]. While telemedicine specifically refers to the remote diagnosis and treatment of patients through telecommunications technology, telehealth covers a wider range of remote health care services, including remote nonclinical services, public health education services, and instruction to medical students [3]. Wearable devices are defined as consumer-facing, sensor-enabled devices that can continuously or intermittently track health-related data such as physical activity, heart rate, or sleep patterns. Examples include smartwatches, fitness trackers, and health-monitoring rings. These tools play a growing role in personal health management and are increasingly integrated into remote care models like telehealth.

While there is increasing attention to adopting advanced technology in health care systems, the gap between the potential of telehealth and its actual adoption needs further exploration. Especially for the aging population, who are not early adopters of technology, it is crucial to understand their preferences and barriers. The Health Information National Trends Survey (HINTS) provides valuable data in this regard. HINTS is a major initiative of the National Cancer Institute that collects data on the American public’s use of health information and technology [4]. The survey contains key issues such as how individuals access and use health information, their perceptions of health-related risks, and their health behaviors and conditions. HINTS 6 (2022) is the sixth cycle of the HINTS program and reflects the evolving landscape of digital health during the postpandemic period. This cycle includes updated questions on telehealth usage, wearable device adoption, and digital health communication, making it particularly well-suited for examining how older adults engage with emerging health technologies. The HINTS 6 survey results have been examined for patterns and predictors of telehealth utilization—a study used logistic regression analysis to reveal significant associations of social determinants, socioeconomic demographics, and health factors with telemedicine services utilization [5]. The study found that despite the widespread use of telemedicine, US adults still prefer in-person visits in the early postpandemic years, especially older adults, who showed a lower inclination toward telemedicine. This insight highlights the current challenge for telehealth utilization for older adults, despite the substantial benefits that broader telehealth services offer older adults, particularly in managing chronic conditions and promoting physical activity. Older adults are defined as individuals aged 65 and above, which aligns with demographic classifications used in national datasets such as HINTS and prior public health literature [4]. This age group is particularly relevant for examining telehealth adoption, as they face increasing health management needs and may benefit most from remote care solutions. However, the reasons behind this slow adoption are not fully understood. Our paper aims to bridge the gap by leveraging the HINTS 6 dataset to conduct a focused analysis of telehealth adoption among the aging population. By examining factors such as demographics, physical activity levels [6-8], health conditions, and technological barriers (use of wearable devices) [9], this study seeks to build a comprehensive understanding of the variables influencing telehealth utilization in older adults, especially the impact of technical barriers. In this study, we use the term “telehealth” to align with the terminology used in the HINTS 6 survey. The survey defines a telehealth visit as “a telephone or video appointment with a doctor or health professional.” While e-health encompasses a broader variety of digital health technologies, our analysis specifically focuses on telehealth usage as defined and measured in the HINTS dataset.

### Aging Population

The phenomenon of an aging population is a relatively recent challenge from a historical perspective [10]. In 1950, no nation had more than 11% of its population aged 65 years and older. By 2000, this figure had risen to 18%, and projections suggest a dramatic increase to 38% by 2050 [11]. The World Health Organization anticipates that by mid-century, the number of individuals aged 65 and older will surpass the number of youths aged 15‐24. This demographic change is a medical and social issue worldwide, with the increase in the aging population affecting the socioeconomic structure and underscoring the need for specialized products and services for older adults [12].

Due to this worldwide challenge, the concept of healthy aging emerges, which emphasizes developing and maintaining functional abilities that promote well-being in older age, as defined by the World Health Organization [13]. This paradigm shift requires a re-evaluation of current health care models that focus not only on disease response but also on the multiple health determinants—behavioral, clinical, social, and environmental—that influence functional decline across the lifespan [14]. The primary goal of this approach is to extend health span rather than lifespan, aiming to reduce morbidity associated with age-related chronic diseases [15].

As life expectancy increases, the prevalence of noncommunicable diseases such as diabetes, renal failure, arthritis, Alzheimer disease, and Parkinson disease among older adults increases, requiring ongoing surveillance and a comprehensive management strategy. This demographic change is accompanied by physical and cognitive decline, increasing the risk of comorbidities and acute medical events such as falls or stroke [16]. Therefore, promoting healthy aging while ensuring the autonomy and safety of older adults at home has become a key social goal.

### Adoption of Wearable Devices

The advent of technology has led to a wide variety of wearable devices that go beyond traditional smartwatches to include innovative smart jewelry designed to be worn around the fingers, ears, or neck [17]. These devices have revolutionized self-tracking, with physical health and activity levels becoming the primary interests of users. In addition, wearable devices enable a broader range of health indicators, including stress levels, emotional well-being, sleep quality, geographic location, nutritional habits, vital signs, and specific disease or symptom tracking [18]. This comprehensive monitoring capability allows health care professionals to have continuous access to the health status of older adults and offers unique opportunities for effective remote care [19], thereby facilitating effective remote care and personalized health management strategies. For example, a smartwatch equipped with a heart rate sensor can alert users and health care providers to irregular heartbeats, suggesting potential arrhythmia or other cardiovascular issues [20]. Similarly, wearable fall detection devices can automatically notify family members or emergency services in the event of a fall, significantly reducing the response time and potentially saving lives [21]. Furthermore, devices tracking sleep patterns can offer valuable data to adjust treatments for insomnia or sleep apnea, contributing to mental well-being [22].

Wearable devices enable older adults to manage their health proactively, continuously track chronic conditions and treatments, and monitor potential safety hazards—all without disrupting daily life [23]. This device not only supports independent living but also improves quality of life. In the health care domain, wearable devices are gradually being integrated into patient care protocols and independent living programs for the aging population [24]. Researchers have explored the utility of wearable devices in forecasting clinical outcomes [25], monitoring patient activity levels, especially in those with chronic conditions like chronic obstructive pulmonary disease [14], predicting patient-reported outcomes such as pain scores [26], monitoring cardiac arrhythmia [27], and tracking the progression of symptoms in neurodegenerative diseases [28]. The growing interest in wearable devices among health care providers, including general practitioners considering prescribing mobile health apps and devices, signals a transformative shift in patient care and contributes to the growth in telehealth [28].

Combined with wearable devices, telehealth has become a key aspect of modern health care, seamlessly extending medical services directly into older adults’ homes. By enabling continuous monitoring and management of chronic conditions, telehealth can reduce the frequency of hospital visits, lower health care costs, and improve the efficiency of health care delivery. In addition, telehealth can significantly improve the accessibility of health care services, ensuring that older people receive timely and appropriate care no matter where they are. The ability to conduct virtual consultations and access health care services from home is particularly beneficial during public health crises, such as the COVID-19 pandemic, when minimizing physical contact is critical [29]. Despite the significant advantages of telehealth, several obstacles hinder its widespread adoption, particularly among the aging population [30]. Digital literacy is a major barrier; many older adults are unfamiliar with the necessary technology, which can limit their access to telehealth services [31]. Privacy and security concerns regarding personal health data transmitted over the internet further exacerbate these challenges [32]. Additionally, the digital divide—a gap in access to digital devices and high-speed internet—disproportionately affects rural and underserved communities, thereby limiting the reach and effectiveness of telehealth solutions.

### Hypothesis

As wearable devices are one of the prominent enablers of at-home health monitoring, we propose that being familiar with wearable devices may increase the adoption of other emerging technologies, including telehealth, particularly among the aging population (age ≥65). This hypothesized relationship stems from the strategy for breaking down technology barriers [33]. Adaptation to one technology can reduce barriers to the adoption of related technologies. Specifically, individuals accustomed to wearable devices may find the transition to telehealth services more intuitive. This relationship may be influenced by demographic factors: a higher level of education, which often correlates with greater openness to technological adoption; higher income ranges, being able to afford such health-related products and services; and the influence of living in rural or urban areas, which could affect the accessibility of in-person clinical care and hence the need for telehealth services. Additional variables that necessitate telehealth service and relate to health awareness include general health and intention to engage in physical exercise. In this study, we will explore the following hypotheses:

1. The use of wearable devices among the aging population is associated with telehealth adoption.
2. Demographic factors (including gender, education, income, and location) are associated with telehealth adoption in the aging population.
3. Demographic factors act as mediating variables, influencing how the use of wearable devices is associated with telehealth adoption.
4. Poor health conditions in the aging population are directly associated with higher telehealth adoption.
5. Health conditions mediate the relationship between the use of wearable devices and the adoption of telehealth among the aging population.
6. An active lifestyle is significantly associated with a higher likelihood of telehealth adoption among the aging population.

## Methods

### Study Design and Dataset

The HINTS survey is conducted every 2 years to gather comprehensive insights into the changing landscape of health communication and information dissemination [4]. HINTS 6 (2022) data were collected from March through November 2022, and it contains responses from 6252 participants. Participants were selected using a random sample of US households, with addresses drawn from the US Postal Service’s Computerized Delivery Sequence File, and respondents were offered a paper survey or web option. This 2-pronged approach helped capture a wide demographic, including digitally literate individuals and those more comfortable with traditional survey methods. To ensure sufficient representation, HINTS incorporates a stratified sample design, oversampling rural populations and older adults to capture a broad range of health behaviors and technology use across different demographic groups.

We used 10 variables from the survey based on the hypotheses; 5 of them are demographic variables. All the variables and related survey questions are summarized in Table 1. Telehealth adoption is defined as the use of telehealth services delivered digitally and virtually, such as video or telephone consultations with health professionals. Responses indicating the use of video, phone calls, or a combination of both were considered evidence of telehealth adoption. The use of wearable devices in the HINTS survey and this study refers to the engagement with electronic devices designed to monitor or track health and physical activity, such as a Fitbit, Apple Watch, or Garmin Vivofit. While wearable devices may collect health data that can support telehealth services, they do not inherently constitute telehealth adoption. Notably, telehealth involves direct communication with health care providers, whereas wearable device use is focused on personal health monitoring.

**Table 1.** Descriptions of variables used in the study from the HINTS 6 survey.

Variable and variable name	Survey item
Age	
AgrGrpB	R1. What is your age?
Telehealth adoption	
ReceiveTelehealthCare	D1. In the past 12 months, did you receive care from a doctor or health professional using telehealth?
OfferedTelehealthOption	D2. In the past 12 months, were you offered the option to have a telehealth visit for any medical care you tried to schedule?
Use of wearable devices	
WearableDevTrackHealth	B8. In the past 12 months, have you used an electronic wearable device to monitor or track your health or activity? For example, a Fitbit, Apple Watch, or Garmin Vivofit.
Health condition	
GeneralHealth	H1. In general, would you say your health is...?
Physical exercise	
TimesModerateExercise	M1. In a typical week, how many days do you do any physical activity or exercise of at least moderate intensity, such as brisk walking, bicycling at a regular pace, and swimming at a regular pace (do not include weightlifting)?
Location	
PR_RUCA_2010	USDA 2010 Primary Rural-Urban Community Area Code
Education	
Education	R7. What is the highest grade or level of education?
Gender	
BirthGender	R2. On your original birth certificate, were you listed as male or female?
Income	
IncomeRanges	R14. Thinking about members of your family living in this household, what is your combined annual income, meaning the total pre-tax income from all sources earned in the past year?

a HINTS: Health Information National Trends Survey.

### Data Processing

The primary outcome considered in this study is the adoption of telehealth among the aging population. The telehealth adoption score was assessed using the following 2 survey items: (1) “In the past 12 months, were you offered the option to have a telehealth visit for any medical care you tried to schedule?” and (2) “In the past 12 months, did you receive care from a doctor or health professional using telehealth?” We formed a composite score to represent the adoption of telehealth based on these 2 survey questions. We assume that the answers “Yes, by video,” “Yes, by phone call,” and “Yes, some by video and some by phone call” are indicative of telehealth use, while “No telehealth visits in the past 12 months” and “I did not try to schedule any medical care in the past 12 months” suggest no use of telehealth. Table 2 shows the telehealth adoption score.

To enhance the analytical clarity and efficiency of our study, we simplified categorical variables related to education level, income ranges, and frequency of moderate exercise. Then, we encoded all categorical variables, including demographic variables, health conditions, and physical exercises.

**Table 2.** Telehealth adoption conditions.

Telehealth adoption conditions	Label
Not offered telehealth and did not adopt	0
Offered telehealth but did not adopt	1
Adopted telehealth	2

To better represent the range of educational attainment in our dataset, we condensed the categories into 4 main levels:

Less than high school (coded to 1): This category combines “less than 8 years” and “8 through 11 years” into a single category, representing individuals who have not completed high school education.

High school plus (coded to 2): This new category merges “12 years or completed high school,” “Post high school training other than college (vocational or technical,” and “Some college” into one. It captures those who have achieved education beyond high school but have not obtained a full college degree.

College graduate (coded to 3): Retained as a distinct category, it signifies individuals who have completed a college degree.

Postgraduate (coded to 4): Also kept separate, indicating individuals with education beyond a college degree.

Income ranges were redefined into 3 broad categories to reflect the socioeconomic status of the study participants effectively: low income ($0-$49,999), middle income ($50,000 -$99,999), and high income ($100,000 or more), encoding as 1, 2, and 3 accordingly. The frequency of moderate exercise was encoded numerically to facilitate quantitative analysis of physical activity patterns among participants. The encoding represents the number of days per week participants engage in moderate exercise, ranging from 0 (no exercise) to 7 (daily exercise).

### Data Analysis

The data analysis used ordinal regression and mediation analysis to examine factors that influence telehealth adoption among the aging population based on the HINTS 6 (2022) dataset. Ordinal regression was used to determine the direct associations between telehealth adoption and independent variables, including wearable device use, demographics (age, gender, education, income, location), health conditions, and physical exercise. The analysis was chosen because the outcome variable—telehealth adoption—is ordinal, represented by 3 levels indicating increasing engagement (no use, offered but not adopted, adopted). This method allows us to estimate the cumulative odds of telehealth adoption across the 3 categories, providing insight into how the predictors influence the likelihood of being in a higher adoption category.

After identifying factors—education, income, and health conditions—that influence telehealth adoption from the ordinal regression, we further decomposed the effect of each factor and conducted a parallel mediation analysis to quantify the mediating effects on the relationship between the use of wearable devices and telehealth adoption. This approach allows us to identify and quantify pathways through which these mediators influence adoption behavior, offering a more nuanced understanding of the mechanisms underlying telehealth adoption. To support this, a conceptual model was developed to illustrate both direct and indirect relationships. Such models are frequently used in behavioral and health policy research to enhance interpretation and effectively communicate findings, particularly when informing intervention design and strategy development.

### Ethical Considerations

This study was a secondary analysis of publicly available data from the HINTS 6 administered by the National Cancer Institute. As such, it is exempt from institutional review board (IRB) review per US federal guidelines on secondary data use [34]. No compensation was provided by the authors for participation, as data collection was performed independently by the HINTS program. This manuscript does not include any identifiable individual participant data or images. Therefore, no consent forms for identifiable data are required.

## Results

### Data Processing and Selection

**Figure 1.**
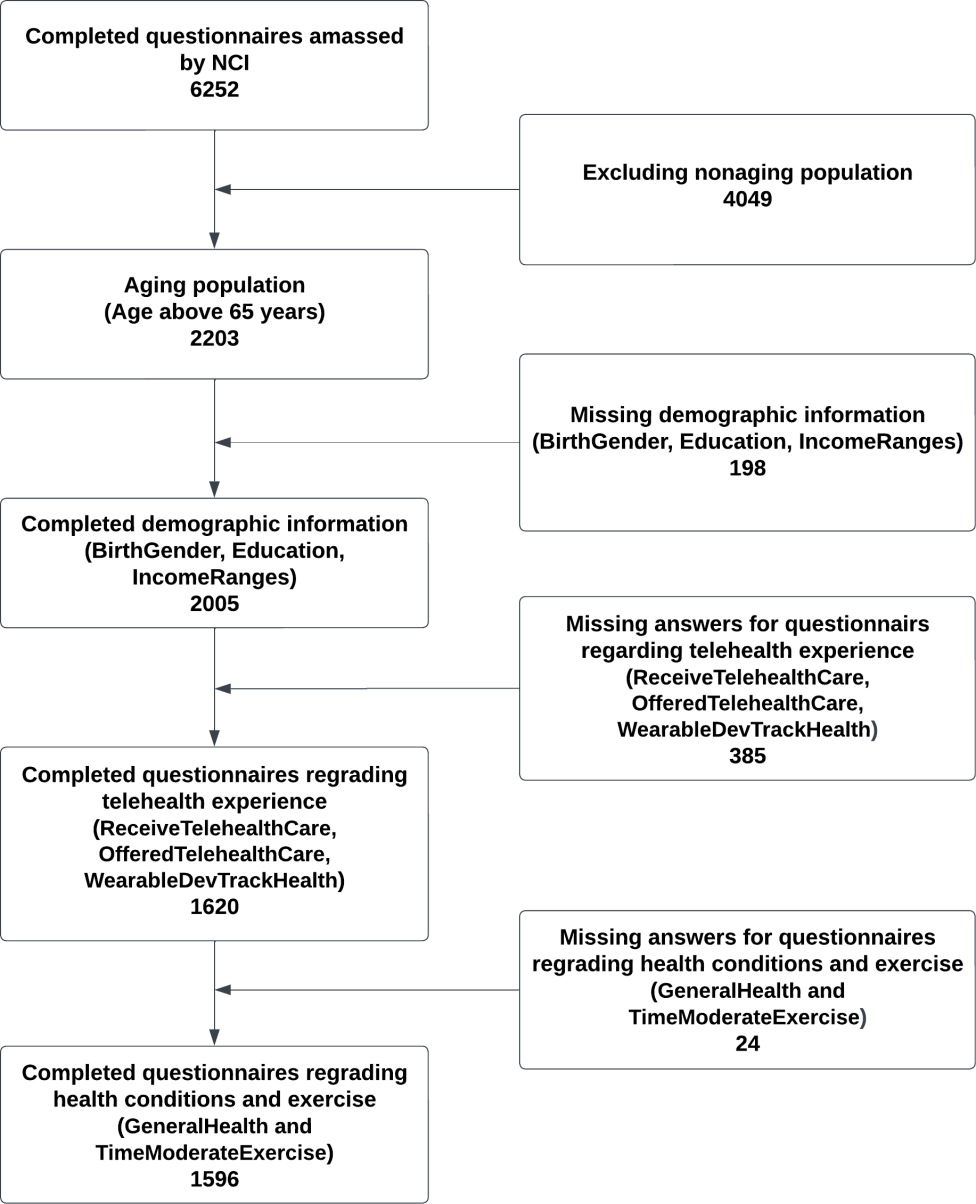
Flow diagram illustrating the selection of survey respondents aged ≥65 years from the Health Information National Trends Survey (HINTS) 6 dataset. NCI: National Cancer Institute.

A total of 6252 surveys were included in the HINTS 6 dataset. For our study, we focused specifically on the aging population, defined as individuals aged 65 years and older. Out of the initial dataset, 2203 participants met this age criterion. After further refinement and excluding incomplete data, the subsequent processing and analysis were conducted on 1596 complete responses. The data selection process is shown in Figure 1.

### Ordinal Regression Analysis

The ordinal regression analysis was performed on the refined HINTS 6 dataset to establish the association between telehealth adoption and various factors, including the use of wearable devices, demographic variables, health conditions, and engagement in physical exercise. The result is shown in Table 3.

**Table 3.** Results from ordinal logistic regression that assesses the association between wearable device use, demographics, and telehealth adoption among older US adults (N=1596).

Factors	Coefficient	*t* value	*P* value	Odd ratios (95% CI)	Reference categories
Use of wearable devices	0.2762	2.2134 (1576)	.03	1.3181 (1.03-1.68)	No
Location	−0.2538	−3.1019 (1576)	.003	0.7758 (0.655-0.918)	Metropolitan
Gender	0.0347	0.3388 (1576)	.74	1.0354 (0.841-1.290)	Female
Income	0.1369	1.8648 (1576)	.07	1.1468 (0.993-1.323)	Low income
Education	0.3113	4.9108 (1576)	<.001	1.3653 (1.206-1.562)	Less than high school
Health condition	−0.2659	−4.5104 (1576)	<.001	0.7665 (0.683-0.860)	Poor
Physical exercise	−0.0376	−1.6056 (1576)	.11	0.9631 (0.920-1.008)	None

The use of wearable devices demonstrated a statistically significant positive association with telehealth adoption (*P*=.03). This finding supports Hypothesis 1 and shows that an increased use of wearable devices is associated with increased adoption of telehealth. Specifically, the odds ratio of 1.3181 suggests that individuals who use wearable devices are approximately 32% more likely to adopt telehealth compared to those who do not.

In addition, the location variable has a statistically significant association with telehealth adoption, with a *P* value of 0.0025. Residents in rural areas have lower levels of telehealth adoption. Education emerged as the most highly significant factor (*P*<.001), with a positive coefficient (0.3113). The odds ratio for education (1.3653) implies that with each increase in education level, the odds of telehealth adoption increase by approximately 36.5%. This strongly supports the notion that educational attainment plays a critical role in health technology adoption.

In contrast, gender does not have a statistically significant effect on the likelihood of adopting telehealth services. Income also did not reach the conventional threshold for significance (*P*=.07), but its marginal association suggests that further investigation should be required to understand income’s role at a more granular level. Given the mixed results among these demographic factors, Hypothesis 2 is partially supported. The factors of gender and income did not show a significant impact, whereas rural location and lower education undermined telehealth adoption.

The self-reported health conditions are inversely associated with telehealth adoption (coefficient=−0.26597, *P*<.001), thereby supporting Hypothesis 4. Physical exercise, representing moderate levels of physical activity, may be negatively associated with telehealth adoption (coefficient=−0.03761, *P*=.11). Thus, Hypothesis 6 is not supported, and instead, an active lifestyle potentially decreases the likelihood of telehealth adoption.

### Mediation Analysis and Conceptual Model Development

After excluding gender and physical exercise due to their lack of significant relationships with telehealth adoption, subsequent correlation analyses were performed on the remaining variables. The correlations between features are moderate to low (<0.5), which is illustrated in Table 4, so keeping them as separate mediators is acceptable.

**Table 4.** Results of correlation analysis between key independent and dependent variables.

Variables	1	2	3	4	5	6
1. Use of wearable devices						
*r*	1	–0.042	0.159	0.188	0.117	0.071
*P* value	—	.09	<.001	<.001	<.001	.004
2. Location						
*r*	−0.042	1	−0.071	−0.089	−0.066	−0.086
*P* value	.09	—	.005	<.001	.009	.001
3. Education						
*r*	0.159	−0.071	1	0.416	0.246	0.131
*P* value	<.001	.005	—	<.001	<.001	<.001
4. Income						
*r*	0.188	−0.089	0.416	1	0.240	0.088
*P* value	<.001	<.001	<.001	—	<.001	<.001
5. Health condition						
*r*	0.117	−0.066	0.246	0.240	1	−0.082
*P* value	<.001	.009	<.001	<.001	—	.001
6. Telehealth adoption						
*r*	0.071	−0.086	0.131	0.088	−0.082	1
*P* value	.004	.001	<.001	<.001	.001	—

Given that our primary outcome focuses on the relationship between the use of wearable devices for health tracking and telehealth adoption. We then explore how demographic factors like education, income, location, and health condition potentially mediate the effect of the use of wearable devices on telehealth adoption. The mediation analysis revealed that the location does not show a significant relationship with wearable device use, and thus, it does not play a mediating role in our model. This finding did not support Hypothesis 3, so the demographic factors, including location, would not moderate the relationship between the use of wearable devices and telehealth adoption in an aging population. Conversely, other factors such as education, income, and health conditions showed mediation effects, thus partially supporting Hypothesis 3 and fully supporting Hypothesis 5. Our mediation analysis highlights that the use of wearable devices is associated with higher telehealth adoption through the mediating effects of education and income. It indicates that individuals with higher education or income are more inclined to adopt wearable technology, which in turn facilitates the adoption of telehealth services.

We constructed a conceptual model in which the use of wearable devices for health tracking is considered an independent variable, while telehealth adoption is considered a dependent variable. Education, income, and health conditions are treated as mediating variables, potentially influencing the core relationship. This model considers the contextual influences of socioeconomic and health conditions and helps demonstrate pathways through which the use of wearable devices may lead to telehealth adoption. Such an approach allows for a more comprehensive understanding of the multifaceted influences on telehealth adoption. Table 5 summarizes the results of the conceptual model analysis, illustrating how each variable interacts within the model:

**Table 5.** Results of the regression analysis that estimate the direct and indirect effects of the wearable device use on telehealth adoption through education, income, and health conditions.

Regression	Estimate	SE	*z* value	*P* value (>|*z*|)
Telehealth adoption~Use of wearable devices (b1)	0.137	0.077	1.785	.07
Education~Use of wearable devices (a1)	0.339	0.054	6.291	<.001
Telehealth adoption~Education (c1)	0.162	0.034	4.772	<.001
Income~Use of wearable devices (a2)	0.347	0.047	7.383	<.001
Telehealth adoption~Income (c2)	0.114	0.039	2.885	.004
Health condition~Use of wearable devices (a3)	0.262	0.059	4.425	<.001
Telehealth adoption∼Health condition (c3)	−0.125	0.032	−3.870	<.001

### Conceptual Model Analysis

The analysis shows that the use of wearable devices for health tracking has a direct effect on telehealth adoption with a positive coefficient (b1=0.137), but this effect is not statistically significant (*P*=.07). The use of wearable devices is positively correlated with education (a1=0.339, *P*<.001), income (a2=0.347, *P*<.001), and health condition (a3=0.262, *P*<0.001). These results show that higher engagement with wearable devices is associated with higher educational attainment, higher income, and better self-reported health. Subsequently, education (c1=0.162, *P*<.001), income (c2=0.114, *P*=.004), and health conditions (c3=−0.125, *P*<.001) have significant effects on telehealth adoption. The results suggest that higher levels of education and income lead to a greater propensity to adopt telehealth services. This result is consistent with the regression and mediation analysis, supporting Hypothesis 2 and partially supporting Hypothesis 3. Conversely, better general health status appears to decrease the likelihood of telehealth adoption, reflecting a possible reduced need for remote health services among healthier individuals, which confirms the testing results of Hypotheses 4 and 5 in the regression and mediation analysis sections.

**Figure 2.**
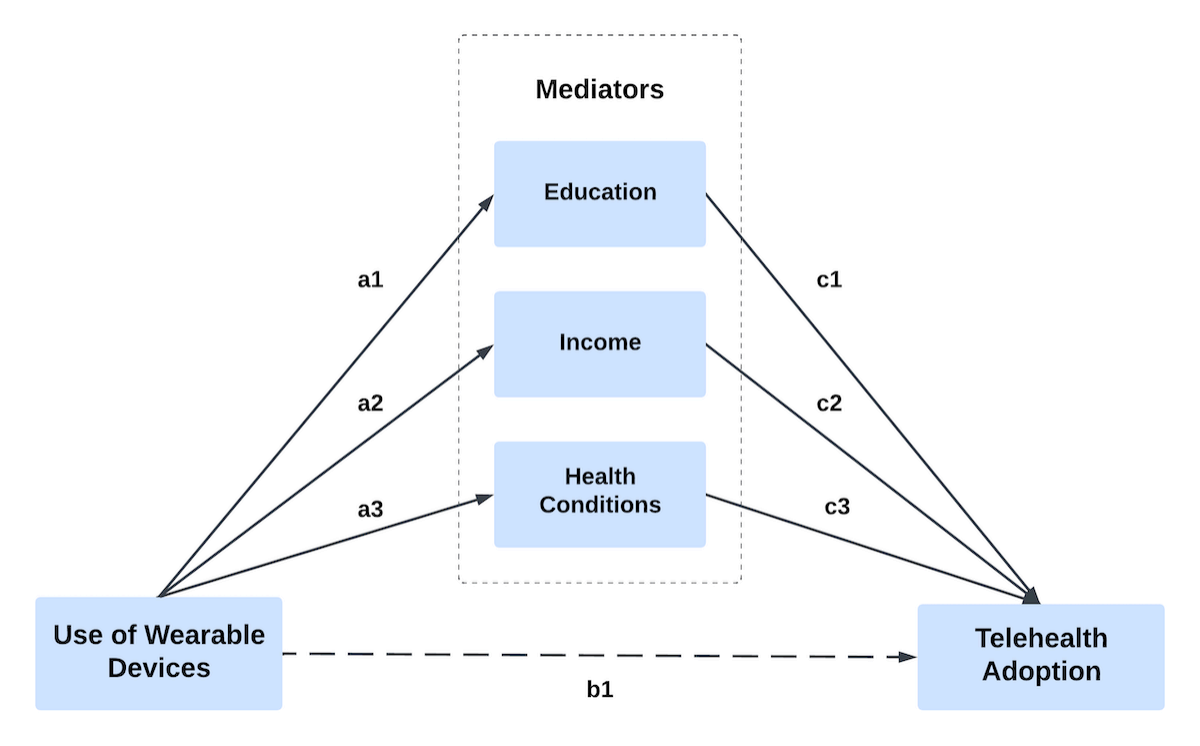
Conceptual model illustrating the relationship between the use of wearable devices and telehealth adoption, with education, income, and health condition as mediators among older US adults.

The mediated effects through education, income, and health conditions are captured by the parameters “indirect1,” “indirect2,” and “indirect3,” respectively, in Table 6, with visualization in Figure 2. The “Estimate” values reported in Table 6 represent standardized indirect effects. These coefficients quantify the extent to which the relationship between wearable device use and telehealth adoption is mediated by education, income, or health conditions. “Indirect1” and “indirect2” positively contribute to telehealth adoption, supporting the beneficial impact of higher education and income facilitated by the use of wearable devices. Conversely, “indirect” shows a negative mediation effect, suggesting that better health conditions actually reduce telehealth adoption. The “total effect” of 0.199 (*P*=.007) encompasses all paths and confirms a significant overall influence of wearable device use on telehealth adoption. To synthesize the statistical results as shown in Table 6 and visualized in Figure 2, we found that demographic factors and health conditions mediate the effect of the use of wearable devices on telehealth adoption among the aging population. This comprehensive model highlights not only isolated effects but also the interconnection of these factors, which is critical to developing targeted strategies to promote telehealth effectively. Table 7 summarizes the outcomes of the hypothesis tests, offering clear insights into the pathways through which the use of wearable devices influences telehealth adoption.

**Table 6.** Total and indirect effects in the conceptual mediation model.

Parameter	Estimate	SE	*z* value	*P* value (>|*z*|)
Indirect1	0.055	0.014	3.809	<.001
Indirect2	0.040	0.015	2.687	.007
Indirect3	−0.033	0.011	−2.915	.004
Total effect	0.199	0.074	2.695	.007

**Table 7.** Summary of hypotheses testing outcomes.

Hypothesis	Path	Coefficient (*P* value)	Outcome
1	Use of wearable devices→Telehealth adoption	0.137 (.07)	Partially supported
2	Education→Telehealth adoption Income→Telehealth adoption	0.162 (<.001) 0.114 (.004)	Partially supported
3	Use of wearable devices→Mediators→Telehealth adoption	Education: 0.055 (<.001) Income: 0.04 (.007)	Partially supported
4	Health conditions→Telehealth adoption	−0.125 (<0.001)	Supported
5	Use of wearable devices→Health conditions→Telehealth adoption	−0.033 (.004)	Supported
6	Physical exercise→Telehealth adoption	**—**	Rejected

## Discussion

### Principal Findings

The mediation analysis conducted in this study aims to explore the dynamics of telehealth adoption among the aging population, specifically related to the use of wearable devices, demographic factors, and health conditions. Hypothesis 1 was supported across both the ordinal regression and mediation analyses. The use of wearable devices showed a direct effect and also influenced telehealth adoption indirectly through income and education. This suggests that familiarity with digital health tools may help older adults navigate other health technologies more confidently. Although wearable use was associated with increased telehealth adoption, its direct effect was not strong in isolation, highlighting the importance of additional influencing factors. Hypothesis 2 was partially supported. Education and rural location were significant predictors, while gender and income did not reach statistical significance in the ordinal regression. However, income did emerge as a significant mediator in the conceptual model, indicating its nuanced role in the adoption process.

One of the most interesting findings is the role of socioeconomic status, as measured by education and income, in influencing telehealth adoption. Consistent with literature suggesting that higher socioeconomic status facilitates better access to technological resources [35], our study finds that higher education and income levels significantly increased the likelihood of telehealth usage for the aging population too, acting as key mediators in the adoption process. Individuals with higher education and income levels are more likely to adopt technologies in general, which encourages the adoption of telehealth services. This also aligns with the finding in [36], which found similar trends in technology adoption among low-income older adults. Additionally, the role of general health conditions inversely affected telehealth adoption, suggesting that individuals in better health are less likely to perceive a need for such services. This reflects findings by Rodríguez-Fernández et al [37], who found that a history of long-term diseases was positively associated with the effective utilization of telehealth among the aging population. Furthermore, some studies have indicated that self-monitoring technologies not only provide patients with greater awareness and understanding of their condition but also are capable of triggering a virtuous circle [38,39]. Importantly, technology acceptance tends to be higher when adopted in the early stages of the disease [40]. Contrary to the hypothesis, our analysis did not identify a significant relationship between physical activity and higher telehealth adoption, indicating that while physically active individuals may be more health-conscious, they do not necessarily see more value in telehealth services. This observation is consistent with the work of Rochester [41], who also reported limited evidence of a relationship between telehealth usage and physical activity levels.

Although geographic location was not included in the conceptual model as a mediator, it emerged as a significant factor in both the ordinal regression and correlation analyses. Rural residency was associated with lower telehealth adoption, highlighting persistent disparities in digital health access across geographic regions [42]. This finding is particularly important in the US context, where rural residents often face greater travel distances to health care facilities but may lack the broadband infrastructure and digital literacy support needed to engage with telehealth services effectively [43].

The conceptual model in this study examines the use of wearable devices as a predictor of telehealth adoption. Although the direction of the effect was positive, the nonsignificant direct effect highlighted the complexity of the relationship. The increasing use of wearable devices by older adults may not be directly related to increased adoption of telehealth due to variables such as personal attitudes toward the technology, perceived ease of use, and perceived usefulness [44]. However, the significant total effect observed when considering both direct and mediated effects confirms that as wearable use increases, the likelihood of telehealth adoption also increases, driven by the mediating effects of education, income, and general health. This combined effect represents the cumulative impact of the various pathways through which the use of wearable devices impacts telehealth and telemedicine adoption. As discussed in the study by Gupta et al [45], the role of both socioeconomic and psychological factors is important in the acceptance of new technologies.

This study used a parallel mediation framework implemented through ordinal regression to estimate direct and indirect effects across multiple mediators. An alternative approach, such as the Preacher and Hayes bootstrapping method [46], provides robust confidence intervals and is well-suited for testing mediation effects in complex models. Incorporating bootstrapping in future analyses would strengthen the statistical inference of indirect pathways and further validate the conceptual relationships identified in this study.

In summary, this study shows significant indirect effects of socioeconomic factors and health conditions on telehealth adoption in the aging population. While the direct impact of wearable device use on telehealth adoption is subtle, its effects are clearly mediated through pathways mediated by education, income, and health status. This complex relationship highlights the need for multifaceted strategies to enhance access to and adoption of telehealth among older adults. The significant role of wearable devices in facilitating telehealth highlights the need for comprehensive strategies to expand access and adoption among older adults. The previous research highlights that most digital health technologies were not designed for the needs of socially disadvantaged groups, such as older adults or those with limited health literacy skills [47]. As a result, these populations often experience reduced access to and engagement with telehealth services. The COVID-19 pandemic accelerated the shift to telehealth and wearable devices but also amplified digital exclusion, especially among those without consistent internet access or familiarity with remote platforms [48]. The concept of the digital divide encompasses more than device ownership—it includes disparities in broadband access, digital skills, and the ability to meaningfully engage with health technologies [49]. Recent evidence during the COVID-19 pandemic suggests that these disparities directly translate into worse health outcomes [50]. For instance, counties with higher levels of digital exclusion experienced higher COVID-19 case and death rates and lower vaccination uptake compared to digitally connected communities [51]. This suggests that digital exclusion limited the ability of individuals to access timely public health information, schedule vaccine appointments, or participate in virtual care and social support networks. These compounding factors raise urgent concerns about digital equity as a social determinant of health. Consequently, there is an urgent requirement for policies that systematically address both technological and socioeconomic barriers to health technology utilization [52]. These measures are critical to ensuring that all segments of the population benefit from advances in health care technologies and services.

### Limitation

This study has several limitations that should be acknowledged. First, there is a potential for self-selection and self-report bias in the survey responses, which could affect the accuracy of reported use of wearable devices, perceived health conditions, and telehealth adoption. Future studies might benefit from using objective data collection methods, such as direct monitoring of wearable device usage and medical records for telehealth adoption. Second, although the study was controlled for several demographic factors and health-related variables, there may be other confounding factors that may influence the relationship between the use of wearable devices and telehealth adoption. For example, technological literacy, personal attitudes toward health, and availability of support for using digital tools may also play an important role. Third, the mediation model assumes a directional relationship based on theoretical frameworks and prior literature. This study is correlational in nature, and causal claims cannot be made. The findings should be interpreted as associations rather than confirmed causal pathways, and future research using longitudinal or experimental designs is recommended to validate the directionality of these relationships. Fourth, given that the survey was distributed via mail with an optional web-based response, participation may have been lower among those with limited access to or comfort with technology, introducing potential response bias. Fifth, the study focused on the aging US population, so the generalizability of the results may be limited. Differences in culture, economics, and health care systems may influence the adoption of telehealth in other regions. For instance, the US health care system is largely privatized and insurance-dependent, with varying levels of access to services based on coverage and cost. This structure can shape how older adults engage with both telehealth and wearable technologies. In contrast, countries with universal health care systems, centralized digital health platforms, or stronger community-based care models may exhibit different drivers of technology use among older adults. It is worth noting that North America has one of the highest adoption rates of wearable devices globally. According to Statista, by the end of 2022, North America had 439 million connected wearable devices, the highest of any region. Projections also show continued growth, with over 90 million US users expected in 2025 across smart bands, smartwatches, and smart scales [53]. This widespread adoption indicates that older adults in the US may already be more familiar with wearable devices, potentially increasing their openness to integrating telehealth solutions as well. Therefore, while the findings provide valuable insights into the American aging population, they may not be directly applicable to populations outside the United States without consideration of these structural and contextual differences.

### Future Direction

Future studies should explore long-term patterns of telehealth adoption and address persistent barriers, particularly in low-income and rural communities. While our study sheds light on structural and demographic influences, it does not directly capture users’ perceived benefits of telehealth. To complement these findings, future research could incorporate validated instruments—such as the Telehealth Usability Questionnaire or surveys based on the Technology Acceptance Model—to assess perceived usefulness, satisfaction, and ease of use among older adults. These tools can help inform more user-centered and effective strategies for promoting telehealth adoption.

### Conclusions

This paper focuses on the influence of wearable usage and the mediating factors of socioeconomic status and health conditions on telehealth adoption among the aging population. The findings indicate that while wearable device use itself may lead to an increase in telehealth adoption, its impact is significantly influenced by factors such as education, income, and health conditions. This trend is consistent beyond the aging population.

Higher levels of education and income were positively associated with telehealth adoption, reflecting greater access and readiness to engage with digital health tools among socioeconomically advantaged individuals. Conversely, better overall health was negatively associated with telehealth adoption, suggesting that healthy older adults may not realize sufficient value in telemedicine services to change their traditional health care engagement habits. This finding suggests that targeted educational workshops may be needed to emphasize the benefits of telehealth not only for immediate care needs but also for preventive health measures.

Overall, the findings provide evidence that telehealth adoption in older adults is shaped by a complex interplay of wearable device use, socioeconomic characteristics, and health status. These insights contribute to a deeper understanding of digital health behaviors in aging populations and align with the study’s objective to investigate key influences on telehealth engagement in this demographic.

## Abbreviations

HINTS: Health Information National Trends Survey
